# Early Response Prediction of Multiparametric Functional MRI and ^18^F-FDG-PET in Patients with Head and Neck Squamous Cell Carcinoma Treated with (Chemo)Radiation

**DOI:** 10.3390/cancers14010216

**Published:** 2022-01-03

**Authors:** Roland M. Martens, Thomas Koopman, Cristina Lavini, Tim van de Brug, Gerben J. C. Zwezerijnen, J. Tim Marcus, Marije R. Vergeer, C. René Leemans, Remco de Bree, Pim de Graaf, Ronald Boellaard, Jonas A. Castelijns

**Affiliations:** 1 Cancer Center Amsterdam, Department of Radiology and Nuclear Medicine, Amsterdam UMC, Vrije Universiteit Amsterdam, De Boelelaan 1117, 1081 HV Amsterdam, The Netherlands; t.koopman@amsterdamumc.nl (T.K.); g.zwezerijnen@amsterdamumc.nl (G.J.C.Z.); jt.marcus@amsterdamumc.nl (J.T.M.); p.degraaf@amsterdamumc.nl (P.d.G.); r.boellaard@amsterdamumc.nl (R.B.); j.castelijns@amsterdamumc.nl (J.A.C.); 2 Department of Radiology and Nuclear Medicine, Amsterdam UMC, University of Amsterdam, Meibergdreef 9, 1105 AZ Amsterdam, The Netherlands; c.lavini@amsterdamumc.nl; 3 Department of Epidemiology and Data Science, Amsterdam UMC, Vrije Universiteit Amsterdam, De Boelelaan 1117, 1081 HV Amsterdam, The Netherlands; t.vandebrug@amsterdamumc.nl; 4 Cancer Center Amsterdam, Department of Radiation Oncology, Amsterdam UMC, Vrije Universiteit Amsterdam, De Boelelaan 1117, 1081 HV Amsterdam, The Netherlands; mr.vergeer@amsterdamumc.nl; 5 Cancer Center Amsterdam, Department of Otolaryngology—Head and Neck Surgery, Amsterdam UMC, Vrije Universiteit Amsterdam, De Boelelaan 1117, 1081 HV Amsterdam, The Netherlands; cr.leemans@amsterdamumc.nl; 6 Department of Head and Neck Surgical Oncology, University Medical Center Utrecht, Heidelberglaan 100, 3584 CX Utrecht, The Netherlands; r.debree@umcutrecht.nl; 7 Department of Radiology, The Netherlands Cancer Institute, Plesmanlaan 121, 1066 CX Amsterdam, The Netherlands

**Keywords:** head and neck, squamous cell carcinoma, functional imaging, MR diffusion weighted imaging, MR dynamic contrast enhanced, PET/CT, radiation therapy/oncology, tumor response, prognosis, outcomes analysis

## Abstract

**Simple Summary:**

Patients with locally-advanced head and neck squamous cell carcinoma (HNSCC) have variable responses to (chemo)radiotherapy. A reliable early prediction of outcomes allows for enhancing treatment efficacy and follow-up monitoring. Early tumoral changes can be captured by functional imaging (DWI/IVIM/DCE/^18^F-FDG-PET-CT) parameters, which allow for the construction of accurate patient-specific prognostic models for locoregional recurrence-free survival, distant metastasis-free survival and overall survival. We also present clinical applicable risk stratification in high/medium/low risks for these patient outcomes. This can enable personalized treatment (adaptation) management early on during treatment, improve counseling and enhance patient-specific post-therapy monitoring.

**Abstract:**

Background: Patients with locally-advanced head and neck squamous cell carcinoma (HNSCC) have variable responses to (chemo)radiotherapy. A reliable prediction of outcomes allows for enhancing treatment efficacy and follow-up monitoring. Methods: Fifty-seven histopathologically-proven HNSCC patients with curative (chemo)radiotherapy were prospectively included. All patients had an MRI (DW,-IVIM, DCE-MRI) and ^18^F-FDG-PET/CT before and 10 days after start-treatment (intratreatment). Primary tumor functional imaging parameters were extracted. Univariate and multivariate analysis were performed to construct prognostic models and risk stratification for 2 year locoregional recurrence-free survival (LRFFS), distant metastasis-free survival (DMFS) and overall survival (OS). Model performance was measured by the cross-validated area under the receiver operating characteristic curve (AUC). Results: The best LRFFS model contained the pretreatment imaging parameters ADC_kurtosis, K_ep_ and SUV_peak, and intratreatment imaging parameters change (Δ) Δ-ADC_skewness, Δ-f, Δ-SUV_peak and Δ-total lesion glycolysis (TLG) (AUC = 0.81). Clinical parameters did not enhance LRFFS prediction. The best DMFS model contained pretreatment ADC_kurtosis and SUV_peak (AUC = 0.88). The best OS model contained gender, HPV-status, N-stage, pretreatment ADC_skewness, D, f, metabolic-active tumor volume (MATV), SUV_mean and SUV_peak (AUC = 0.82). Risk stratification in high/medium/low risk was significantly prognostic for LRFFS (*p* = 0.002), DMFS (*p* < 0.001) and OS (*p* = 0.003). Conclusions: Intratreatment functional imaging parameters capture early tumoral changes that only provide prognostic information regarding LRFFS. The best LRFFS model consisted of pretreatment, intratreatment and Δ functional imaging parameters; the DMFS model consisted of only pretreatment functional imaging parameters, and the OS model consisted ofHPV-status, gender and only pretreatment functional imaging parameters. Accurate clinically applicable risk stratification calculators can enable personalized treatment (adaptation) management, early on during treatment, improve counseling and enhance patient-specific post-therapy monitoring.

## 1. Introduction

The standard treatment of patients with locally advanced head and neck squamous cell carcinoma (HNSCC), is an intensive combination of chemo- and radiotherapy (CRT), which is unfortunately associated with high morbidity (e.g., functional loss) and low overall survival. Due to tumoral heterogeneity, HNSCC comprises a spectrum of tumoral phenotypes, with variable responses to treatment. The early identification of responders to CRT within two weeks after treatment initiation, provides a clinical opportunity before the initiation of irradiation side-effects to consider de-escalation (e.g., radiotherapeutic dose reduction or target volume adaptation) [1]. In contrast, non-responders can benefit from treatment intensification (radiotherapy dose escalation [2], hypoxia modification [3], radio-sensitizers [4]) or switching to surgical treatment [5]).

Potential prognostic tumor-specific characteristics can be captured non-invasively, by anatomical and functional imaging techniques [5,6]. First, ^18^F-FDG-PET assesses glucose metabolism [7]. Secondly, DW-MRI is quantified by calculating the apparent diffusion coefficient (ADC) [8]. Thirdly, an extension of DWI is the intravoxel incoherent motion (IVIM), assessing both the diffusion and perfusion fraction [9]. Finally, dynamic contrast enhancement (DCE) MRI measures tissue perfusion and vessel permeability [10]. All of the aforementioned imaging techniques showed a prognostic value in the prediction of treatment response and patients outcomes [7,8,9,10].

As previously shown, in addition to the pretreatment parameters [6], early anatomical and functional intratumoral changes within two weeks after treatment initiation can also provide important (in)dependent prognostic parameters [5]. Tumor physiology differs markedly, resulting in considerable disparities in how tumors react to the early effects of CRT. This complex interplay of multiple early changing tumor characteristics [11] can be captured in multivariate prediction models. Only one previous study [12] described the prognostic value of some univariate pretreatment parameters in a small cohort, without multivariate analysis or validation. Until now, the combination of multimodality functional imaging parameters capturing (in)dependent early tumoral changes, and the construction of accurate multivariate prediction models, has not yet been assessed.

The aim of this prospective study is to investigate the prognostic accuracy of the combination of functional imaging parameters, including (DW-, IVIM-, DCE-MRI and ^18^F-FDG-PET), acquired pretreatment within 10 days after the initiation of curative (chemo)radiotherapy, and to construct accurate prognostic models and risk stratification calculators in patients with advanced-stage HNSCC. Our study investigates whether multiparametric functional MRI and ^18^F-FDG-PET can predict an early response in patients with head and neck squamous cell carcinoma treated with (chemo)radiation.

## 2. Materials and Methods

### 2.1. Patient Selection

This prospective single-center study, was approved by our local ethical committee (Trial NL3946, NTR4111, 2013-191). Written informed consent was obtained from all patients. Previously untreated histologically-proven HNSCC patients treated with curative (chemo)radiotherapy, were consecutively included between 2013 and 2019 and underwent pretreatment and early intratreatment (10 days after starting treatment), including ^18^F-FDG-PET/low-dose CT, DW- and DCE-MRI. The exclusion criteria were: nasopharyngeal tumors, age < 18 and inadequate image quality (this will be discussed in a later Section of the paper). The adherence of all patients to have pretreatment and intratreatment imaging has been previously reported [13], whereas, in this paper, we report on the early tumoral changes and prognosis. Within five weeks after baseline imaging, treatment was initiated consisting of pre-determined radiotherapy (7 weeks, 70 Gy in 35 fractions) with/without concomitant chemotherapy (3 weekly 100 mg/m^2^ cisplatin), or weekly cetuximab (400 mg/m^2^ loading dose, followed by 7 weekly infusions of 250 mg/m^2^). Some patients underwent an accelerated radiotherapy scheme (6 weeks, 70 Gy in 35 fractions). For oropharyngeal tumors, the HPV status was determined by p16 immunostaining, followed by DNA-PCR on p16 immuno-positive cases.

### 2.2. Imaging

MRI was performed on a 3.0T MR scanner (Ingenuity, Philips Healthcare, Best, The Netherlands) utilizing a 16-channel H&N coil. DWI was performed by fat-suppressed single shot spin-echo echo-planar imaging; TR = 500 ms; TE = 105 ms; echo-planar imaging factor = 35; field of view = 230 × 230 mm; slice thickness = 2 mm; intersection gap = 0.3 mm; matrix = 128 × 128 and receiver bandwidth = 2735.7 Hz per pixel. A total of 10 b-values were used: 0/10/25/50/75/150/300/500/750/1000 s/mm^2^. The ADC map was produced by vendor-provided software.

DCE-MRI was performed by 3 dimensional T1-weighted fat-field echo (FFE); TR/TE = 4.8/2.4 ms; flip angle = 12; FOV = 230 × 230 × 180 mm; matrix = 144 × 144; 75 dynamic acquisitions of 4.16 s and signal averages = 2. An intravenous bolus injection of 0.2 mL/kg of body weight Gd-DOTA (Dotarem, Guerbet, Villepinte, France) was administered after 3 dynamic acquisitions (3 mL/s followed by 25 mL saline flush). The dynamic scan was preceded by 5 scans with the same parameters as the DCE-MRI scan with varying flip angles (2°/5°/10°/15°/20°). This was performed to estimate the quantitative native T1 maps, which were used to convert the signal intensity of the DCE scan into a contrast agent concentration curve, which was used for calculating DCE-derived parametric maps [14].

^18^F-FDG-PET/low-dose CT was performed according to EANM guidelines 2.0 on an EARL accredited Gemini TF-PET/CT (Philips Healthcare, Best, The Netherlands) [15]. The low-dose CT parameters were 120 kV and 30 mAs. Whole body ^18^F-FDG-PET/CT was performed in an arms down position, from the mid-thigh-to-skull vertex, 60 min after intravenous administration of 2.5 MBq/kg ^18^F-FDG, 2 min/bed position. ^18^F-FDG-PET images were reconstructed by vendor-provided reconstruction protocol, with photon attenuation correction, matrix size = 144 × 144 and voxel size = 4 × 4 × 4 mm. Post-reconstruction resolution was 6.75 mm, full width at half maximum.

### 2.3. Exclusion Criteria

MRI scans were excluded if it was not possible to extract quantitative parameters; for example, due to excessive movement or failure to successfully fit the concentration time curves to obtain PKM (Pharmaco Kinetic Model) parameter values in more than 30% of voxels in the ROI. Fit failure occurred mainly due to noise. No patients were excluded due to the low PET image quality.

### 2.4. Delineation

**Figure 1:**
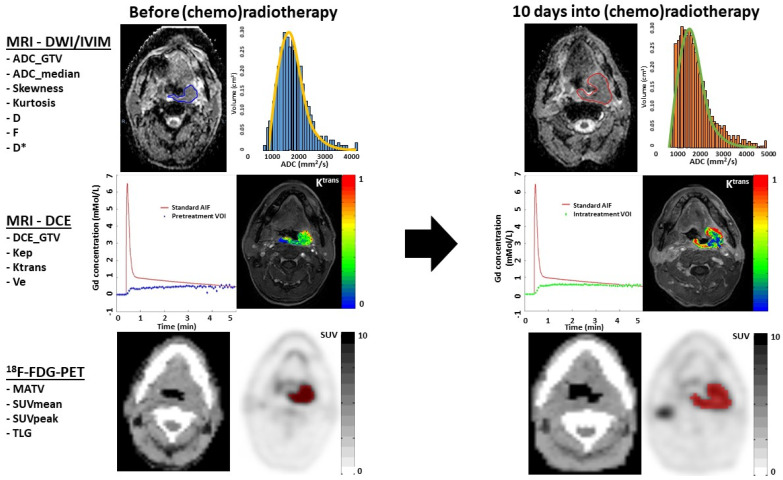
Overview of ADC, IVIM, DCE and FDG-PET imaging acquisition in a patient with left tonsillar carcinoma before and 10 days into (chemo)radiotherapy, and with whom locoregional failure occurred. The upper row shows the ADC map on which the tumor is delineated, in order to extract DWI and IVIM parameters. The subtle spatial mismatch was due to a slightly different angulation of the neck. The ADC histogram shows high pretreatment positive ADC_skewness (blue histogram), and an increase towards a higher intratreatment ADC_skewness (orange histogram). Furthermore, a high pretreatment ADC_kurtosis was associated with LRF (orange line). The middle row shows the population-based arterial input function (AIF) and a tumor concentration time curve. The images are DCE images at the 75 temporal phase, on which a colored functional map of the parameter K^trans^ is superimposed in the delineated tumor. The color scale shows the range between 0 and 1 mMol/L. The ^18^F-FDG-PET image in the lowest row shows the tumor delineation (red ROI) on the attenuation-corrected ^18^F-FDG-PET image (black/white SUV scale ranges between 0 and 10), with a threshold of >50% SUV_peak and in anatomical correlation with a diagnostic CT scan.

Whole-lesion delineation (Figure 1) was manually performed by two independent observers (P.d.G., J.A.C.) on the ADC and DCE image. In this paper, T1, STIR and T2 sequences were used for anatomical correlation, with knowledge of TNM stage and tumor location, but blinded for treatment outcomes. Tumors were delineated on DWI-, IVIM- and DCE-MRI using VELOCITY software (Varian, Palo Alto, CA, USA). DCE-MRI delineation was performed on a contrast-enhanced volume on the 75th dynamic acquisition. The regions delineated on the ADC maps were used to quantify, by region, the ADC and IVIM parameters D, D* and f. ^18^F-FDG-PET/CT delineation was conducted by semi-automatic delineation, by a nuclear medicine specialist (G.J.C.Z.), with 50% of the tumor-specific SUV_peak threshold corrected for local-background uptake [16].

### 2.5. Feature Extraction

Imaging parameters were extracted from the whole-lesion primary tumor ROIs of each observer. The gross tumor volume (GTV) was extracted from the ADC (ADC_GTV), and ADC_skewness and ADC_kurtosis were calculated for each ROI on each parametric map.

The IVIM feature extraction of the perfusion fraction (f), perfusion coefficient (D*) and diffusion coefficient (D) was performed using *MATLAB* R2019a software [17], after motion correction, in order to reduce artifacts.

DCE-MRI analysis was processed with in-house built software (Dynamo; [14]). Quantitative pharmacokinetic model (PKM) analysis was performed by the 2-compartment Tofts model [10] with a population averaged arterial input function (AIF) [18]. The following quantitative parameters were extracted from each voxel: DCE_GTV; K^trans^ (transfer rate of contrast agent from plasma to extravascular, extracellular space); V_e_ (fractional volume of extravascular extracellular space) and K_ep_ (transfer rate of contrast agent from extravascular, extracellular space to plasma). From these PKM parameters, the median values were calculated over delineated 3-dimensional ROIs.

^18^F-FDG-PET/CT in-house built software (accurate; [15]) automatically calculated the whole-lesion metabolic tumor volume (MATV), SUV_mean, SUV_peak (spherical VOI of 1 mL positioned to yield average) and total lesion glycolysis (TLG = SUV_mean × MATV).

In 43 of the 57 patients, the pretreatment parameters (ADC_GTV/DCE_GTV/K_ep_/K^trans^/V_e_/MATV/SUV_mean/SUV_peak/TLG) were previously reported [6]. In the current paper, we report on the pretreatment, intratreatment and delta parameters in multivariate prognostic models and the risk stratification for locoregional recurrence-free survival (LRFS), distant metastasis-free survival (DMFS) and overall survival (OS) prediction.

### 2.6. Statistical Analysis

The mean of two ROI (one by each observer) median parameters per patient in the primary tumor were used as final parameters for statistical analyses (Table S1). The median was used as the statistical representation for each individual parameter, due to the skewed parameter distribution. The fractional changes in the parameters from pretreatment (delta (Δ = (x − pretreatment)/pretreatment)) were calculated for each patient, where x is the second scan (intratreatment) and the pretreatment is the first scan.

Firstly, a prognostic analysis was performed (Mann–Whitney U test), by single univariate parameters predicting responders/non-responders, 2 year LRFFS, DMFS and OS. Furthermore, a prognostic univariate Cox regression analysis was conducted (significance threshold, *p* < 0.05).

Secondly, a prognostic multivariate Cox regression analysis was performed for all the parameters for each modality separately and corrected for the clinical parameters (Table 1) (significance threshold, *p* < 0.05). Subsequently, a prognostic LASSO Cox regression analysis was conducted. The model performance was measured by the cross-validated Harrell’s C index, by repeated cross-validation with 5 folds and 500 repeats.

Separately, a prognostic LASSO logistic regression analysis was performed to fit a prediction model for a 2 year LRFFS, DMFS and OS. Predictive performance was measured by the cross-validated area under the receiver operating characteristic curve (AUC), by repeated cross-validation with 5 folds and 500 repeats.

Based on the best prediction models, risk calculators were constructed, and risk groups were identified by dividing patients into 3 groups (low risk (<33%)/medium (33–66%)/high (≥66%)) for patient outcomes. Log-rank tests and Kaplan–Meier curves were used to analyze the risk differences between groups.

## 3. Results

### 3.1. Patient Characteristics

**Figure 2:**
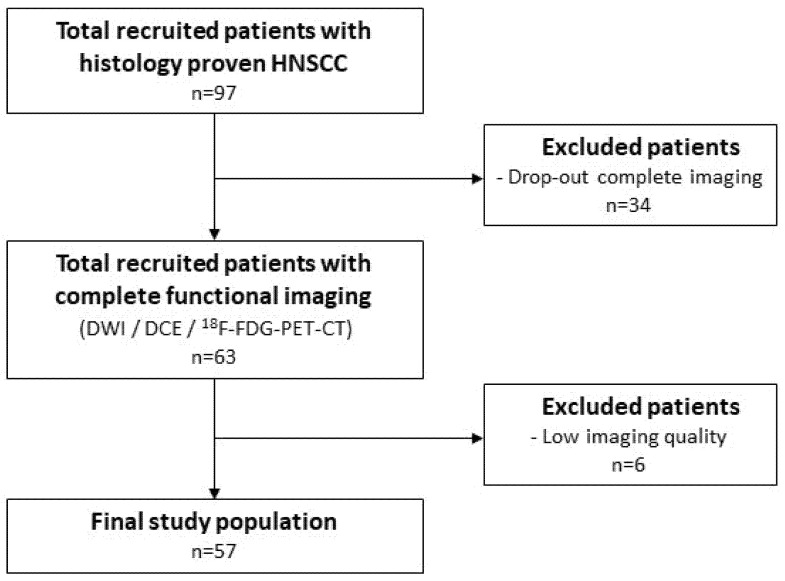
Flowchart of patient inclusion.

Between 2013 and 2019, 97 histological-proven HNSCC patients were recruited [13], of which 63 patients were recruited with complete functional imaging (Figure 2).

A total of 6 patients were excluded due to low imaging quality. The final study population (Table 1) consisted of 57 patients with a hypopharyngeal (*n* = 12) or oropharyngeal (*n* = 45) tumor. A total of 20 (44%) patients within the oropharyngeal tumor subgroup were HPV positive.

A total of 20 patients (35%) had bulky T2-staged tumors with at least N1 disease (stage III) and 37 (65%) T3–4 T-staged tumors). A total of 21 patients (37%) had low N-stage tumors (N0–N1) and 36 (63%) had advanced N-staged (N2–N3) tumors.

A total of 49 patients (86%) received concurrent cisplatin-based chemoradiotherapy. A total of 4 patients (7%) received weekly cetuximab with concurrent radiotherapy (70 Gy) and 3 patients underwent the accelerated radiotherapy scheme (5%). A total of 4 patients (7%) received radiotherapy only. The mean follow-up was 31 months (IQR 18.4–38.7). A total of 18 patients (38.3%) developed a locoregional recurrence, among whom 8 patients (44%) underwent salvage surgery as a secondary treatment. A total of 20 patients (35%) developed distant metastasis. A total of 17 (29.8%) patients died during follow-up, all deaths being related to HNSCC. Among the 45 oropharyngeal HNSCC patients (OPSCC), 13 patients died (4 males (31%)) and there were 26 HPV-negative patients (19 males (73%)).

### 3.2. Observer Variations

When testing the difference within the same parameters resulting from the ROI delineation of the two observers (interobserver agreement, Table S1), the correlation ranged from excellent (r > 0.8) to high (r = 0.6–0.8), except for intratreatment K^trans^ (*p* = 0.001), which showed a moderate correlation (r = 0.55).

### 3.3. Parameter Correlations

The correlations between all the different parameters (resulting from an average of the ROIs delineated by the two observers), always resulted in r values lower than 0.9 (not tabulated). Therefore, we included all parameters in the prognostic analyses.

### 3.4. Prognostic Parameters

In Tables S2–S6, significant differences of both univariate analyses (*p* < 0.05), of clinical and imaging parameters, were summarized between patients with locoregional control (LRC) versus locoregional failure (LRF), no distant metastasis (no DM) versus distant metastasis (DM) and alive versus death.

**Figure 3:**
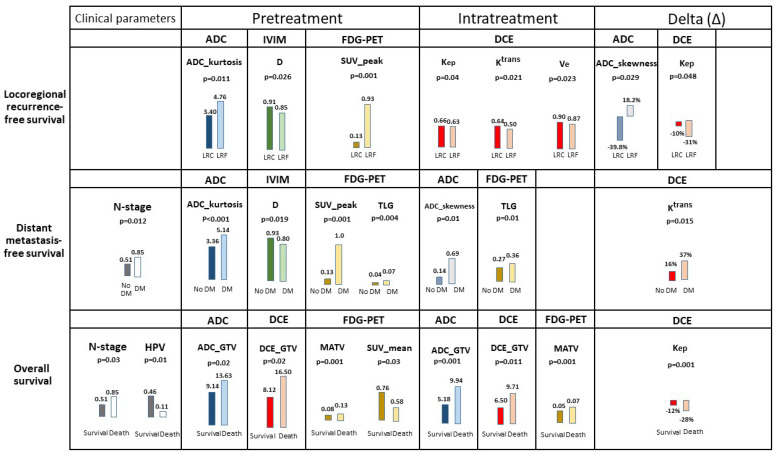
The median of significant multivariate prognostic pretreatment, intratreatment and delta-parameters per single imaging modality for locoregional recurrence-free survival, distant metastasis-free survival and overall survival (see Tables S2 and S3 for the complete tables). HPV-negative tumors were scored with the number 0 and HPV tumors with the number 1.

In the multivariate LRFFS analysis (Figure 3), pretreatment parameters ADC_kurtosis, D and SUV_peak, and intratreatment parameters K_ep_, K^trans^, V_e_, TLG, Δ-ADC_skewness and Δ-K_ep_, were significantly prognostic (*p* < 0.05) (Table S4).

In the multivariate DMFS analysis (Figure 3), pretreatment N-stage, ADC_kurtosis, D, SUV_peak, TLG, intratreatment ADC_skewness, TLG and Δ-K^trans^, were significantly prognostic (*p* < 0.05) (Table S5).

In the multivariate OS analysis (Figure 3), N-stage, HPV status, pretreatment ADC_GTV, DCE_GTV, MATV, SUV_mean, intratreatment ADC_GTV, DCE_GTV, MATV and Δ-K_ep_ were significantly prognostic (*p* < 0.05) (Table S6).

### 3.5. Prognostic Models

**Figure 4:**
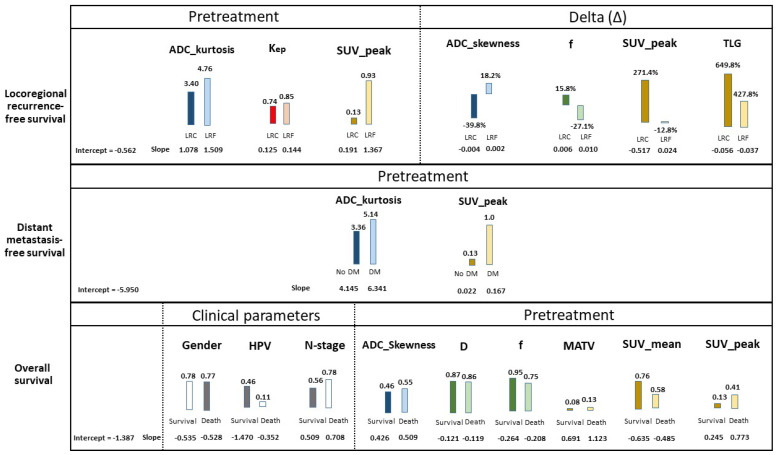
Significant multivariate prognostic pretreatment, intratreatment and delta parameters of all imaging techniques combined for locoregional recurrence-free survival, distant metastasis-free survival and overall survival (See Tables S2 and S3 for the complete tables). Overall, for each patient outcome, the intercept and the slopes per median parameter was shown. The median slopes were found lower in patients with locoregional control (LRC) than locoregional failure (LRF), lower in no distant metastasis (no DM) than distant metastasis (DM), and lower in survival than death, which resulted in a lower risk for an adverse outcome. HPV-negative tumors were marked with a 0, and HPV-positive tumors with a 1. Gender was marked with a 0 for females and 1 for males.

The best logistic regression prediction model for LRFFS (Table 2) was based on pretreatment and delta parameters, which resulted in the highest prognostic accuracy (cross-validated AUC = 0.81, Figure S2). This model included the pretreatment variables ADC_kurtosis, K_ep_ and SUV_peak, and the delta parameters Δ-ADC_skewness, Δ-f, Δ-SUV_peak and Δ-TLG (Figure 4, Table S7). The prognostic accuracy for LRFFS did not improve when including clinical parameters. The best LRFFS Cox regression model showed a cross-validated C index = 0.75 (Table 3 and Table S8).

The best logistic regression model for DMFS (cross-validated AUC = 0.88, Table 2) was based on pretreatment parameters only (ADC_kurtosis and SUV_peak) (Figure 4, Table S7). The best DMFS Cox regression model showed a cross-validated C index= 0.79 (Table 3 and Table S8).

The best logistic progression prediction model for OS (cross-validated AUC = 0.82, Table 2) was based on the clinical parameters of gender, HPV status, N-stage and pretreatment parameters only (ADC_skewness, D, f, MATV, SUV_mean and SUV_peak) (Figure 4, Table S7). The best OS Cox regression model showed a cross-validated C index = 0.75 (Table 3 and Table S8).

### 3.6. Risk Stratification

**Figure 5:**
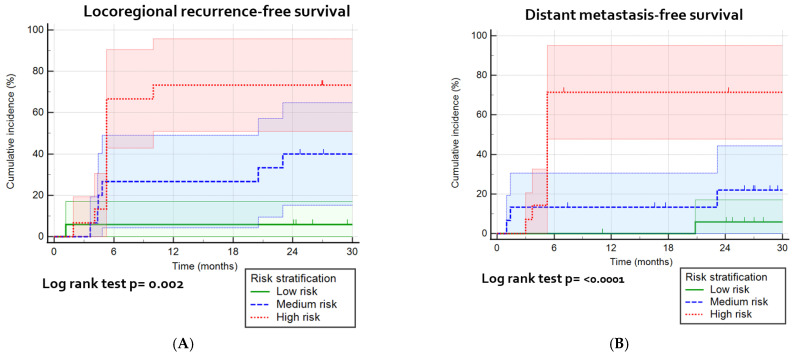

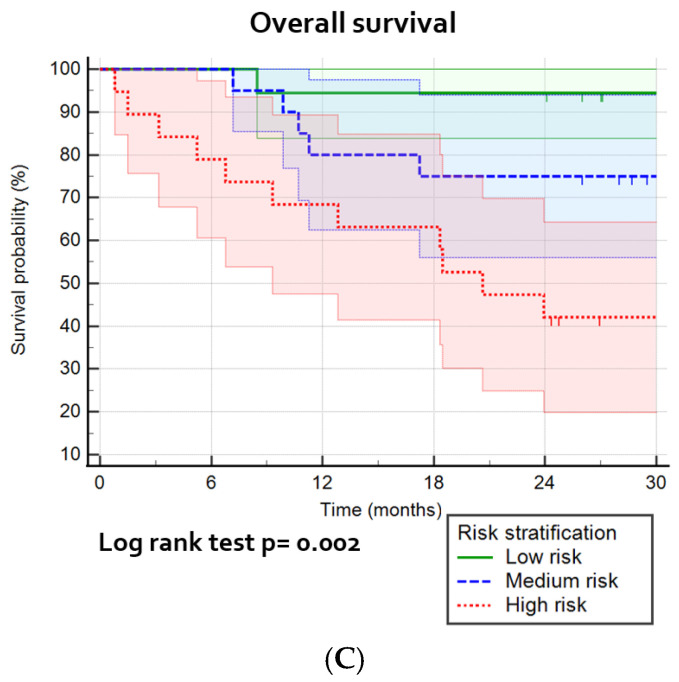
Kaplan–Meier survival curves and the log-rank test for the most optimal prognostic models: (**A**) the combination of pretreatment, intratreatment and delta parameters for the cumulative incidence of locoregional recurrence, divided into high/medium/low risk groups; (**B**) prediction model with pretreatment parameters for the cumulative incidence for distant metastasis, divided into high/medium/low risk groups; and (**C**) prediction model with pretreatment parameters prognostic for overall survival, divided into high/medium/low risk groups.

The risk stratification calculators were presented in Table 4, which shows the included parameters in the best prediction models. A division of patients into three risk groups, based on predicted probabilities (high/medium/low risk) (Figures S3–S5), shows a significant prediction of LRFFS (*p* = 0.002), DMFS (*p* < 0.0001) and OS (*p* = 0.002; Figure 5). The high risk sensitivity and specificity was for the best models for LRFFFS 0.61 and 0.83, for DMFS 0.71 and 0.82, and for OS 0.65 and 0.78, respectively (not tabulated).

## 4. Discussion

In patients with advanced-stage HNSCC treated with curative chemoradiotherapy, tumoral characteristics, such as cell density and necrosis, and vascular and metabolic demand and supply, were captured by imaging parameters (DWI-, IVIM-, DCE-MRI and ^18^F-FDG-PET parameters). Early treatment effects were captured by assessing the difference between early intratreatment and baseline parameters. The combination of these functional imaging parameters (with clinical parameters) showed (in)dependent prognostic values, and allowed for the construction of accurate clinical applicable prediction models and risk calculators, which offer the prospect to enhance personalized treatment management early on during treatment. However, intratreatment imaging showed an added value in the prediction of LRFFS only, and did not add to the DMFS and OS prediction models.

In the current study on locoregional failure-free survival prediction, the multivariable prognostic model, using a combination of parameters that were not previously described, revealed a high accuracy (AUC = 0.81). We found that low Δ-ADC_skewness (decrease) was prognostic for LRC, which confirmed the results of the previous work [19,20]. This can be explained by a shift of the histogram peak and tail from low ADC values (positive skewness; peak on left side) in viable tumors with densely packed cells, toward high ADC values (negative skewness; peak on right side), due to cellular death [19,20]. Furthermore, low pretreatment kurtosis (broad distribution of voxel values) was associated with LRC, which was previously described [19,20]. This can reflect that large viable solid tumor components (low ADC values; negative right-skewed histograms) and small necrotic areas [21] have the potential to shift towards a normally distributed histogram, due to the early effects of treatment (cell death) [19]. In contrast, LRF was associated with high pretreatment ADC_kurtosis (narrow distribution near the mean ADC voxel values), with larger stromal components and necrotic areas, and showed an increase in positive skewness. This can be caused by treatment effects, with increasing hypoxic radiotherapy-resistant necrotic areas and less viable tumors, non-accessible by therapy [21].

Furthermore, in current LRFFS predictions, an increase in Δ-f was prognostic for LRC. Increased blood flow (high f) can reflect a beneficial oxygen supply, enhancing radiosensitivity [22]. This was in line with lower tumor blood volume (ΔTBV) in LRF [23,24], possibly causing an insufficient response to chemoradiotherapy. Moreover, low pretreatment K_ep_ was associated with LRC. In this group, sufficiently vascularized tumors might have balanced vascular permeability (K^trans^), interstitial volume (V_e_) and reflux, back into the vascular system (K_ep_). In contrast, in LRF, insufficient vascularization, possibly due to accelerated imbalanced angiogenesis, might have resulted in higher vascular leakage (K^trans^), higher interstitial volume (V_e_) and/or high K_ep_, which can result in a greater resistance to radiotherapy (e.g., larger hypoxic/necrotic areas) [25]. Although K^trans^ was significantly prognostic in several studies [26,27], the current study shows no significant prognostic value of K^trans^ in the multivariate models.

Finally, in the current LRFFS prediction, a larger increase in TLG, lower pretreatment SUV_peak with a high delta SUV_peak showed to be prognostic for LRF. This was confirmed in a previous study [28], which found a higher SUV_peak (cut-off ≥ 14.1) associated with LRF. Enhanced metabolic activity was found to be correlated with the overexpression of HIF-1a, which characterizes cellular responses to hypoxic stress [29] and is a prognostic factor for LRF, which can imply a more aggressive phenotype (higher grade) [30].

In the current DMFS prediction model, a low pretreatment ADC_kurtosis and a low SUV_peak were associated with improved distant metastasis-free survival (AUC = 0.88), which has never been previously described. This can reflect that tumors with a normally distributed ADC histogram can be less likely to metastasize. Furthermore, only other pretreatment metabolic PET parameters were previously described as being associated with the occurrence of DM, such as high SUVmax, MATV [31,32] and TLG [32]. This indicated that high tumoral metabolism reflects a tumoral phenotype that is prone to metastasize.

In the current OS prediction model (AUC = 0.82), HPV positivity is associated with a favorable OS, which is widely confirmed in the literature [33]. Furthermore, the female gender was found to be favorably prognostic for survival. This can be due to the low female-to-male ratio (1:3) for HPV-negative HNSCC incidence, which has been previously described [33]. Furthermore, low pretreatment ADC_skewness, high D and f were found prognostic for survival. This can imply that the majority of tumoral ADC values are moderately low (low positive skewness), with sufficient diffusion (high D) and vascularity (high f) being associated with good overall survival. In contrast, high pretreatment MATV and SUV_peak and low pretreatment SUVmean were associated with adverse OS. These findings were confirmed by a pretreatment multivariate PET-only study [34], and 26 univariate PET-only studies [34], which showed that high TLG and MTV are associated with adverse OS. This can represent an aggressive highly metabolic tumoral phenotype, resulting in adverse OS. In contrast, a low SUVmean can represent a heterogeneous tumor phenotype, suggesting the presence of areas of low FDG uptake.

The clinical applicable prognostic risk stratification calculators presented in the current study, allow for patient-specific risk prediction for LRFFS, DMFS and OS. However, it should be validated in future studies. Another scoring system was previously described, based on study-related cut-off values for TLG and tumor uniformity [35], but without validation or identification of clinical usable risk groups.

The prediction models presented in this paper, have a high accuracy for LRFFS, DMFS and OS, and can be used for clinical decision making. The obtained information from these prediction models can be used for the intensification or de-escalation of treatment, or even change of primary treatment modality to improve survival and quality of life where possible. Although treatment adaptation may not always be possible, it also provides important information for counseling and more personalized follow-up treatment, allowing for the early detection of recurrent and residual disease.

This study has been limited to the heterogeneity in the limited patient sample size, in which the tumor location, HPV status and tobacco use, varied among patients. Although these parameters were combined in the LASSO regression analyses, which corrected these differences and made the models more generalizable, a risk for overfitting might have remained. Furthermore, some post-processing steps in DCE-MRI were necessary, due to either movement or excessive noise, which harbors the risk of interobserver variability, even though a high overall interobserver correlation was found. Furthermore, in this study, we used a population-averaged AIF. This is a possible cause of the misrepresentation of the quantitative PKM parameters. We observed that the tumoral concentration time curve was sometimes higher than in the standard AIF, resulting in a calculated V_e_ higher than 1, in some patients. We assume that this systematic error was consistent within the same patient and, therefore, has not affected the change of the specific parameter. Furthermore, only the pre-intratreatment imaging parameters of the primary tumors were assessed to enhance parameter comparability, whereas locoregional recurrence was examined as patient outcomes. Therefore, locoregional recurrence might have developed in a lymph node metastases with (not measured) functional imaging parameters different from the primary tumor. Another limitation is the variable interval of pretreatment and intratreatment imaging. In patients with longer intervals between pretreatment imaging and the start of treatment, the tumor can become larger and parameters (e.g., imaging parameters) can change, regardless of treatment effect. Finally, another limitation of this study may be the survival data based on a follow-up of 2 years. However, for the assessment of LRFFS, DMFS and OS, other studies [36,37] used a similar follow-up time and showed that the majority of events occurred within 2 years.

## 5. Conclusions

Early intratreatment tumoral changes can be captured by functional imaging parameters. However, intratreatment and delta parameters showed a prognostic value in the prediction of LRFFS only, and did not add to the DMFS and OS prediction models. The most accurate prognostic models for LRFFS are a combination of pretreatment, intratreatment and delta imaging parameters. The presented clinical applicable risk calculators, after future validation, can enable personalized treatment management early on during treatment, allowing for individualized treatment adaption (intensification, de-escalation or modality change). The most accurate prognostic model for DMFS and OS was based on pretreatment parameters, in which, for OS prediction, also HPV-status and gender were prognostic. These prognostic models can enhance survival and quality of life, improved counseling and enhanced patient-specific post-therapy-monitoring.

## Figures and Tables

**Table 1:** Patient characteristics.

Patient Characteristics and Events			
Age at baseline imaging		Follow-up after treatment	
Median (IQR *)	63 (56.5–67)	Follow-up in months (IQR *)	30.7 (17.8–38.7)
Sex		Treatment	
Female	16	Chemoradiotherapy	53
Male	42	Cisplatin	49
Tobacco use		Cetuximab	4
None (%)	21 (36.8)	Radiotherapy only	4
Smoker (%)	36 (63.2)	No. tumor-related events	
HPV positive (%)	20 (44.4) †	Locoregional recurrence	18
Primary tumor location		Distant metastases	20
Oropharynx	45	Tumor-related death	17
Hypopharynx	12		
T stage (*n* = 58)			
2	18		
3	15		
4	25		
N stage (*n* = 58)			
0	13		
1	8		
2	34		
3	2		

* Interquartile range; † measured in the oropharynx.

**Table 2:** Prognostic models (Lasso logistic regression) of the 15 imaging features (Table S4) without and with clinical features (Table S2), predicting locoregional recurrence-free survival, distant metastasis-free survival and overall survival, with the amount of patients (Table S7). The amount of features and the area under the curve (AUC) with the standard deviation (SD) is tabulated.

Logistic Regression Models								
Outcome	Features	Patients	Imaging Features			Clinical Parameters + Imaging Features		
			Features	AUC	SD	Features	AUC	SD
Locoregional recurrence-free survival	PRE	47	15	0.79	0.16	22	0.79	0.16
	INTRA	47	15	0.49	0.09	22	0.47	0.09
	Delta	47	15	0.76	0.14	22	0.77	0.14
	ALL	47	45	0.81	0.14	52	0.80	0.15
Distant metastasis	PRE	57	15	0.79	0.12	22	0.84	0.18
	INTRA	57	15	0.63	0.15	22	0.81	0.17
	Delta	57	15	0.52	0.12	22	0.82	0.15
	ALL	57	45	0.86	0.15	52	0.88	0.13
Overall survival	PRE	57	15	0.62	0.18	22	0.82	0.12
	INTRA	57	15	0.46	0.13	22	0.64	0.15
	Delta	57	15	0.48	0.11	22	0.66	0.15
	ALL	57	45	0.53	0.17	52	0.69	0.16

**Table 3:** Prognostic models (Lasso Cox regression) of the 15 imaging features (Table S3) without and with clinical parameters (Table S2) combined, predicting locoregional recurrence-free survival, distant metastasis-free survival and overall survival (Table S8). The amount of features and the area under the curve (AUC) with the standard deviation (SD) is shown.

Cox Regression Models								
Outcome	Features	Patients	Imaging Features			Clinical Parameters + Imaging Features		
			Features	C index	SD	Features	C index	SD
Locoregional recurrence-free survival	PRE	57	15	0.70	0.18	22	0.69	0.15
	INTRA	57	15	0.48	0.10	22	0.48	0.10
	Delta	57	15	0.75	0.14	22	0.73	0.15
	ALL	57	45	0.72	0.15	52	0.72	0.16
Distant metastasis	PRE	57	15	0.79	0.13	22	0.75	0.15
	INTRA	57	15	0.64	0.16	22	0.64	0.14
	Delta	57	15	0.52	0.14	22	0.58	0.16
	ALL	57	45	0.77	0.14	52	0.75	0.14
Overall survival	PRE	57	15	0.65	0.18	22	0.75	0.15
	INTRA	57	15	0.48	0.12	22	0.55	0.14
	Delta	57	15	0.49	0.10	22	0.66	0.10
	ALL	57	45	0.53	0.16	52	0.62	0.15

**Table 4:** The risk-of-locoregional-recurrence calculator, which can be used in clinical practice to calculate the risk per specific patient of locoregional recurrence during the follow-up time of 2 years. The yellow boxes can be filled in with the specific patient data in order to calculate the risk of locoregional recurrence, distant metastasis or death, in which gender is either 1 (male) or 0 (female). HPV status is either 1 (positive) or 0 (negative) and N-stage is either 0 (stage 0–1) or 1 (stage 2–3). The risk-of-metastasis calculator, which can be used in clinical practice to calculate the risk per specific patient of metastasis during the follow-up time of 2 years. The yellow boxes are filled in with the single patient data (with a large tumor) in order to calculate the risk of metastasis. The risk-of-death calculator, which can be used in clinical practice to calculate the risk per specific patient of death during the follow-up time of 2 years. The yellow boxes can be filled in with the single patient data in order to calculate the risk of death.

Locoregional Recurrence Risk Calculator				Metastasis Risk Calculator				Death Risk Calculator			
Predictor	Fill-In	Formula	Result	Predictor	Fill-In	Formula	Result	Predictor	Fill-In	Formula	Result
**PRE-ADC_kurtosis**	x	0.317326	**A**	**PRE-ADC_kurtosis**	**x**	1.233603	**A**	**Gender**	**x**	−0.685436	**A**
**PRE-K_ep_**	x	0.168844	**B**	**PRE-SUV_peak**	x	0.167255	**B**	**HPV**	**x**	−3.196345	**B**
**PRE-SUV_peak**	x	1.469971	**C**					**N-stage**	**x**	0.908168	**C**
**Δ-ADC_skewness**	x	0.010764	**D**					**PRE-ADC_skewness**	**x**	0.925759	**D**
**Δ-f**	x	−0.036035	**E**					**PRE-D**	**x**	−0.138775	**E**
**Δ-SUV_peak**	x	−0.190617	**F**					**PRE-f**	**x**	−0.277885	**F**
**Δ-TLG**	x	−0.008579	**G**					**PRE-MATV**	**x**	8.640004	**G**
								**PRE-SUV_mean**	x	−0.836001	**H**
								**PRE-SUV_peak**	x	1.885920	**I**
**Linear probability**	Y = −2.173498 + (Sum **A + B + C + D + E + F + G**)			**Linear probability**	Y = −5.950312 + (Sum **A + B**)			**Linear probability**	Y = −1.387043+ (Sum **A + B + C + D + E + F + G + H + I**)		
**Probability formula**	**1/(1 + exp(−Y)) =**			**Probability formula**	**1/(1 + exp(−Y)) =**			**Probability formula**	**1/(1 + exp(−Y)) =**		
**Risk**			**%**	**Risk**			**%**	**Risk**			**%**

## Data Availability

Data generated or analyzed during the study are available from the corresponding author by request.

## Supplementary Materials

The following are available online at https://www.mdpi.com/article/10.3390/cancers14010216/s1, Table S1: Interobserver correlation of quantitative MRI parameters, Table S2: Clinical parameters per patient outcome, Table S3: Primary tumor imaging parameters per patient outcome, Table S4: Univariate and multivariate analysis predicting locoregional recurrence-free survival, Table S5: Univariate and multivariate analysis predicting distant metastasis-free survival, Table S6: Univariate and multivariate analysis predicting overall survival, Table S7: Logistic regression prognostic models of primary tumors, Table S8: Cox regression prognostic models of primary tumors, Figure S1: Overview of analyses, Figure S2: The area under the curves for LRFFS, DMFS and OS, Figure S3: Locoregional recurrence-free survival curves per risk group, Figure S4: Distant metastasis-free survival curves per risk group, Figure S5: Overall survival curves per risk group.

## Abbreviations

AIF	Arterial input function
AJCC	American Joint Committee on Cancer
D	Pure diffusion coefficient [mm^2^/s]
D*	Pseudo-diffusion coefficient [mm^2^/s]
DCE	Dynamic contrast enhanced
DMFS	Distant metastasis-free survival
DWI	Diffusion-weighted imaging
f	Perfusion fraction
GTV	Gross tumor volume
HNSCC	Head and neck squamous cell carcinoma
IVIM	Intravoxel incoherent motion
K_ep_	The rate constant for transfer of contrast agent from extravascular, extracellular space to the plasma [min^−1^]
K^trans^	The rate constant for transfer of contrast agent from plasma to extravascular, extracellular space [min^−1^]
LRC	Loco-regional control
LRF	Loco-regional failure
LRFFS	Loco-regional failure-free survival
MATV	Mean of the active tumor volume
OPSCC	Oropharyngeal squamous cell carcinoma
OS	Overall survival
SUV_mean	Mean of SUV included in the VOI
SUV_peak	Sphere of 8 voxels around the voxel with the highest SUV in the delineated VOI
TLG	Total lesion glycolysis (SUVmean × MATV)
TLG40%	Total lesion glycolysis, including voxels with SUV higher than 40% of SUV_peak
V_e_	Fractional volume of the extravascular extracellular space
