# Supplementary material for: Early Response Prediction of Multiparametric Functional MRI and 18F-FDG-PET in Patients with Head and Neck Squamous Cell Carcinoma Treated with (Chemo)Radiation
Source: Cancers (Basel). 2022 Jan 3;14(1):216. doi: 10.3390/cancers14010216 (PMC8750157; doi:10.3390/cancers14010216)
Supplement: Supplementary file 1 [file cancers-14-00216-s001.zip › cancers-1486308-supplementary.pdf]

cancers MDPI

Supplementary Materials: Early Response Prediction of Multiparametric Functional MRI and ^{18}F-FDG-PET in Patients with Head and Neck Squamous Cell Carcinoma Treated with (Chemo)Radiation

Roland M. Martens, Thomas Koopman, Cristina Lavini, Tim van de Brug, Gerben J.C. Zwezerijnen, J. Tim Marcus, Marije R. Vergeer, C. René Leemans, Remco de Bree, Pim de Graaf, Ronald Boellaard and Jonas A. Castelijns

Table S1. Interobserver correlation quantitative MRI-parameters.

		PRETREATMENT				INTRATREATMENT			
		Primary tumor							
		Observer 1	Observer 2	Difference	Correlation	Observer 1	Observer 2	Difference	Correlation
		Mean (SD)	Mean (SD)	Wilcoxon Signed rank test	Pearsons r	Mean (SD)	Mean (SD)	Wilcoxon Signed rank test	Pearsons r
DWI	ADC_GTV(x103cm^3)	10.504 (10.055)	10.051 (8.991)	0.069	0.944	10.871 (0.104)	10.927 (0.105)	0.476	0.997
	ADC_median(x103mm^2/s)	0.105 (0.182)	0.105 (0.180)	0.292	0.994	0.126 (0.199)	0.126 (0.181)	0.687	0.971
	ADC_skewness	0.368 (0.508)	0.396 (0.519)	0.870	0.847	0.222 (0.452)	0.233 (0.463)	0.851	0.921
	ADC_kurtosis	3.769 (1.21)	3.732 (1.318)	0.515	0.910	3.295 (0.806)	3.318 (0.837)	0.771	0.869
IVIM	D(mm^2/s)	0.009 (0.001)	0.009 (0.140)	0.633	0.986	0.001 (<0.001)	0.001 (<0.001)	0.674	0.986
	f(mm^2/s)	0.099 (0.061)	0.100 (0.062)	0.870	0.991	0.101 (0.046)	0.102 (0.052)	0.307	0.960
	D*(mm^2/s)	0.006 (0.002)	0.007 (0.003)	0.163	0.994	0.006 (0.001)	0.006 (0.002)	0.077	0.988
DCE	DCEGTV(cm3)	12.035 (9.490)	11.414 (9.300)	0.002	0.985	10.099 (8.714)	9.910 (8.897)	0.064	0.872
	Kep(min^{-1})	0.830 (0.298)	0.745 (0.287)	<0.001	0.827	0.714 (0.261)	0.716 (0.283)	0.463	0.563
	K^{trans}(min^{-1})	0.535 (0.215)	0.513 (0.221)	0.002	0.808	0.646 (0.266)	0.606 (0.236)	0.013	0.373
	V_e	0.080 (0.195)	0.745 (0.228)	<0.001	0.775	0.983 (0.293)	0.944 (0.293)	0.841	0.698

The mean, standard deviation (SD). the Wilcoxon signed rank test and the Pearson interobserver correlation test of all quantitative MRI-parameters (DWI, IVIM and DCE) calculated for the two observers. Overall, there were significant differences between the delineations of both observers among the DCE parameters for primary tumor and DWI, IVIM and DCE for LNM. The correlation ranged from excellent ($r > 0.8$) to good ($0.6 < r < 0.8$), except for PT Kep and Ve (r=0.563 and r=373. respectively).

cancers MDPI

Table S2. Clinical parameters per patient outcome.

Clinical parameters (mean±SD)	Locoregional recurrence-free survival			Distant metastasis-free survival			Overall survival		
	Responders	Non-responders	P-value	No DM	DM	P-value	Survival	Death	P-value
Gender	0.744±0.44	0.833±0.38	0.459	0.676±0.47	0.95±0.22	0.020	0.778±0.43	0.769±0.43	0.944
Age	60.564±8.69	64.667±5.62	0.049	60.784±9.13	63.85±5.08	0.188	61.718±8.70	62.167±6.58	0.661
T-stage	0.718±0.46	0.50±0.51	0.112	0.784±0.42	0.40±0.50	0.004	0.590±0.50	0.778±0.43	0.171
N-stage	0.590±0.50	0.722±0.46	0.339	0.514±0.51	0.85±0.37	0.013	0.564±0.50	0.778±0.43	0.123
HPV	0.308±0.47	0.444±0.51	0.319	0.243±0.43	0.55±0.51	0.022	0.462±0.51	0.111±0.32	0.011
Location primary tumor (PT)	0.18±0.39	0.278±0.46	0.402	0.243±0.43	0.15±0.37	0.414	0.205±0.41	0.222±0.43	0.884
Smoking	0.667±0.48	0.556±0.51	0.423	0.758±0.43	0.50±0.52	0.320	0.564±0.50	0.778±0.43	0.087

The mean and standard deviation of the clinical parameters per responders versus non-responders, no distant metastasis (No DM) versus distant metastasis (DM), survival versus death, with the Mann-Whitney U test. The significant differences between these groups is marked green ($p<0.05$). In this analysis all the analysed observables were made binary: For gender, value 1 represents male, 0 is women. For T-stage $0<T<1$ (T=0 when stage is 1 or 2, and T=1 when Stage is 3 or 4). For Nodal stage N=0 => stage 0 and 1, N=1= stage 2 and 3, $0<N<1$. For location PT: 0=oropharynx, 1=Larynx. For smoking: 1= smoker (20 package/years)+ stopped shorter than 2 years)

Table S3. Primary tumor imaging parameters per patient outcome

Primary tumor

Locoregional recurrence-free survival

	Parameters	Pretreatment			Intratreatment			Delta		
	(median±stddev)	Responders	Non-responders	P-value *	Responders	Non-responders	P-value *	Responders	Non-responders	P-value *
DWI	ADC_GTV(xE3cm3)	7.396±7.3	10.199±5.7	0.196	9.143±8.01	4.662±8.1	0.583	0.054±0.7	-0.438±0.7	0.004
DWI	ADC_median(xE3mm2/s)	1.100±0.2	0.957±0.1	0.181	1.1277±0.2	1.149±0.2	0.148	0.221±0.2	0.224±0.1	0.775
DWI	ADC_skewness	0.238±0.4	0.584±0.4	0.554	0.150±0.4	0.675±0.4	0.039	-0.398±6.3	0.182±12.9	0.313
DWI	ADC_kurtosis	3.397±0.8	4.764±1.0	0.007	3.165±0.8	4.032±0.7	0.035	-0.046±0.2	-0.216±0.3	0.429
IVIM	D(mm2/s) xE3	0.913±0.1	0.853±0.1	0.042	1.036±0.2	1.055±0.3	1.000	0.158±0.3	0.278±0.2	0.120
IVIM	f(mm2/s) xE1	0.935±0.6	0.862±0.8	0.511	1.013±0.4	0.849±0.3	0.220	0.158±1.0	-0.271±0.7	0.101
IVIM	D*(mm2/s) xE2	0.620±0.1	0.620±0.4	1.000	0.624±0.1	0.575±0.1	0.431	-0.008±0.3	-0.102±0.3	0.526
DCE	DCEGTV(cm3)	7.620±7.7	12.150±8.3	0.393	6.750±6.7	6.945±8.8	0.399	-0.253±0.4	-0.140±0.5	0.678
DCE	Kep(min−1)	0.744±0.3	0.854±0.3	0.018	0.659±0.2	0.630±0.3	0.526	-0.101±0.3	-0.308±0.4	0.096
DCE	K^{trans}(min−1)	0.510±0.2	0.575±0.3	0.229	0.633±0.2	0.495±0.4	0.437	0.174±0.6	0.141±0.7	0.759
DCE	V_e	0.700±0.2	0.698±0.2	0.393	0.898±0.4	0.870±0.3	0.686	0.402±0.4	0.309±0.6	0.662
^{18}F-FDG-PET	MATV(cm3) xE-5	0.068±0.1	0.089±0.04	0.074	0.056±0.1	0.050±0.0	0.913	-0.259±0.6	-0.441±0.3	0.483
^{18}F-FDG-PET	SUV_mean(Bq) xE-6	0.819±0.3	0.757±0.2	0.525	0.574±0.2	0.719±0.2	0.380	-0.163±2.4	-0.056±0.7	0.709
^{18}F-FDG-PET	SUV_peak(Bq) xE-1	0.125±0.3	0.932±0.4	0.001	0.579±0.3	0.770±0.3	0.254	2.714±3.2	-0.128±0.2	0.002
^{18}F-FDG-PET	TLG(Bq*cm3)	0.039±0.1	0.068±0.04	0.083	0.275±0.4	0.359±0.1	1.000	6.498±16.5	4.278±5.4	0.013

Distant metastasis-free survival

	Parameters	Pretreatment			Intratreatment			Delta		
	(median±stddev)	No DM	Distant metastasis	P-value *	No DM	Distant metastasis	P-value *	No DM	Distant metastasis	P-value *
DWI	ADC_GTV(xE3cm3)	7.352±6.6	10.199±6.5	0.101	7.462±8.5	4.662±7.1	0.581	0.026±0.8	-0.543±0.4	0.073
DWI	ADC_median(xE3mm2/s)	1.101±0.2	0.896±0.1	0.019	1.299±0.2	1.096±0.1	0.021	0.195±0.2	0.224±0.1	0.893
DWI	ADC_skewness	0.136±0.4	0.584±0.3	0.002	0.139±0.4	0.691±0.2	0.000	-0.539±11.3	0.182±0.4	0.027
DWI	ADC_kurtosis	3.357±0.6	5.14±0.8	0.000	3.165±0.8	4.032±0.7	0.005	-0.006±0.2	-0.216±0.2	0.209
IVIM	D(mm2/s) xE3	0.933±0.2	0.801±0.1	0.010	1.059±0.2	0.981±0.2	0.292	0.188±0.3	0.278±0.2	0.358
IVIM	f(mm2/s) xE1	0.607±0.6	0.862±0.8	1.000	1.027±0.5	0.843±0.3	0.160	-0.011±1.0	-0.114±0.8	0.593
IVIM	D*(mm2/s) xE2	0.639±0.1	0.571±0.5	0.249	0.624±0.2	0.545±0.1	0.098	-0.008±0.3	-0.079±0.3	0.960
DCE	DCEGTV(cm3)	7.620±6.4	12.325±10.0	0.270	7.210±6.8	6.600±9.2	0.558	-0.231±0.5	-0.199±0.3	0.920
DCE	Kep(min−1)	0.744±0.3	0.843±0.3	0.482	0.648±0.3	0.655±0.3	0.828	-0.152±0.3	-0.158±0.5	0.664
DCE	K^{trans}(min−1)	0.550±0.2	0.485±0.3	0.091	0.625±0.3	0.525±0.3	0.867	0.161±0.5	0.368±0.8	0.045
DCE	V_e	0.700±0.2	0.704±0.2	0.132	0.880±0.3	0.932±0.3	0.894	0.391±0.4	0.473±0.6	0.026
^{18}F-FDG-PET	MATV(cm3) xE-5	0.071±0.1	0.089±0.1	0.110	0.055±0.1	0.050±0.0	0.639	-0.283±0.6	-0.441±0.2	0.789
^{18}F-FDG-PET	SUV_mean(Bq) xE-6	0.720±0.3	0.762±0.2	0.750	0.559±0.2	0.72±0.2	0.032	-0.170±2.3	-0.056±0.7	0.492
^{18}F-FDG-PET	SUV_peak(Bq) xE-1	0.131±0.3	0.996±0.4	0.001	0.579±0.3	0.869±0.3	0.052	-0.065±2.9	-0.128±2.8	0.402
^{18}F-FDG-PET	TLG(Bq*cm3)	0.039±0.1	0.068±0.1	0.132	0.270±0.2	0.359±0.4	0.215	5.625±15.8	4.28±5.7	0.323

Overall survival

	Parameters	Pretreatment			Intratreatment			Delta		
	(median±stddev)	Survival	Death	P-value *	Survival	Death	P-value *	Survival	Death	P-value *
DWI	ADC_GTV(xE3cm3)	9.140±6.4	13.625±12.1	0.149	5.182±7.1	9.937±14.5	0.216	-0.099±0.8	0.001±0.4	0.718
DWI	ADC_median(xE3mm2/s)	1.048±0.2	1.035±0.1	0.236	1.198±0.2	1.323±0.2	0.371	0.224±0.2	0.206±0.1	0.836
DWI	ADC_skewness	0.459±0.4	0.554±0.4	0.271	0.233±0.4	0.351±0.6	0.606	-0.19±10.2	-0.510±2.2	0.823
DWI	ADC_kurtosis	3.492±1.0	4.143±0.8	0.973	3.330±0.8	3.149±0.8	0.449	-0.086±0.2	-0.075±0.3	0.863
IVIM	D(mm2/s) xE3	0.874±0.1	0.862±0.2	0.372	1.034±0.2	1.068±0.2	0.850	0.261±0.3	0.191±0.3	0.159
IVIM	f(mm2/s) xE1	0.945±0.7	0.751±0.4	0.503	0.896±0.4	0.953±0.6	0.918	-0.023±1.0	0.570±1.0	0.731
IVIM	D*(mm2/s) xE2	0.63±0.3	0.54±0.1	0.175	0.590±0.1	0.603±0.2	0.918	-0.058±0.3	0.184±0.2	0.460
DCE	DCEGTV(cm3)	8.115±7.4	16.50±12.0	0.192	6.503±6.8	9.705±11.0	0.103	-0.223±0.4	-0.087±0.4	0.295
DCE	Kep(min−1)	0.754±0.3	0.794±0.3	0.979	0.658±0.2	0.517±0.3	0.011	-0.118±0.3	-0.283±0.3	0.059
DCE	K^{trans}(min−1)	0.512±0.3	0.471±0.3	0.559	0.634±0.2	0.458±0.4	0.045	0.261±0.5	0.116±0.7	0.328
DCE	V_e	0.700±0.2	0.673±0.2	0.619	0.889±0.3	0.935±0.3	0.619	0.402±0.5	0.527±0.5	0.514
^{18}F-FDG-PET	MATV(cm3) xE-5	0.077±0.1	0.130±0.1	0.049	0.050±0.1	0.07±0.1	0.180	-0.387±0.5	-0.267±0.4	0.744
^{18}F-FDG-PET	SUV_mean(Bq) xE-6	0.762±0.3	0.577±0.2	0.001	0.688±0.2	0.510±0.3	0.159	-0.080±2.1	-0.016±2.8	0.449
^{18}F-FDG-PET	SUV_peak(Bq) xE-1	0.133±0.4	0.406±0.3	0.331	0.734±0.3	0.479±0.2	0.110	-0.074±3.0	0.392±2.2	0.139
^{18}F-FDG-PET	TLG(Bq*cm3)	0.046±0.1	0.036±0.1	0.630	0.359±0.3	0.357±0.9	0.945	5.098±14.5	3.688±8.5	0.630

* Mann-Whitney U analysis

The median of the primary tumor imaging parameters per patient outcome for locoregional recurrence-free survival, distant metastasis-free survival and overall survival. The significant differences between these groups is marked green ($p<0.05$).

Table S4. Univariate and multivariate analysis predicting locoregional recurrence-free survival.

Locoregional recurrence-free survival										
		PRETREATMENT			INTRATREATMENT			DELTA		
	n=57 patients	Univariate*	Multivariate analysis		Univariate*	Multivariate analysis		Univariate*	Multivariate analysis	
	Parameters	p-value	p-value	HR (95%CI)	p-value	p-value	HR (95%CI)	p-value	p-value	HR (95%CI)
Clinical parameters	Gender	0.441	-							
	Age	0.141	-							
	T-stage	0.203	-							
	N-stage	0.268	-							
	HPV	0.710	-							
	Location PT	0.378	-							
	Tobacco usage	0.325	-							
DWI	ADC_GTV (cm^3)	0.846	-		0.501	-	-	0.154	-	
	ADC_median ($x10^3$ mm^2/s)	0.138	-		0.122	-		0.892	-	
	ADC_Skewness	0.600	-		0.219	-		0.029	0.029	0.958 (0.923-0.996)
	ADC_Kurtosis	0.011	0.011	1.883 (1.16-3.06)	0.358	-		0.457	-	
IVIM	D (xE3 mm^2/s)	0.050	0.026	0.018 (0.001-0.61)	0.780	-		0.336	-	
	f (x10 mm^2/s)	0.217	-		0.196	-		0.126	-	
	D* (xE2 mm^2/s)	0.270	-		0.359	-		0.259	-	
DCE	DCE_{GTV} (cm^3)	0.726	-		0.593			0.608	-	
	K_{ep} (min^{-1})	0.025	-		0.774	0.040	0.43E-4 (3.561E-9- 0.508)	0.288	0.048	0.003 (0.000-0.962)
	K^{trans} (min^{-1})	0.099	-		0.817	0.021	235 (2.1 -26297)	0.834	-	
	V_e	0.448	-		0.298	0.023	0.023(0.001-0.565)	0.711	-	
18F-FDG-PET	MATV (cm^3)	0.962	-		0.550	-		0.460		
	SUV_mean (Bq)	0.455	-		0.837	-	-	0.150	-	
	SUV_peak (Bq)	0.001	0.001	10.5 (2.66-41.2)	0.534	-		0.070	-	
	TLG (Bq x cm^3)	0.679	-		0.358	0.049	0.004 (0.00-1.015)	0.144	-	

Univariable (*) and multivariable analysis of functional imaging parameters per modality predicting locoregional recurrence-free survival. Significantly prognostic parameters are marked green.

cancers MDPI

Table S5. Univariate and multivariate analysis predicting distant metastasis-free survival.

Distant metastasis-free survival										
		PRETREATMENT			INTRATREATMENT			DELTA		
	n=57 patients	Univariate	Multivariate analysis		Univariate	Multivariate analysis		Univariate	Multivariate analysis	
	Parameters	p-value*	p-value**	HR (95%CI)	p-value*	p-value**	HR (95%CI)	p-value*	p-value**	HR (95%CI)
Clinical parameters	Gender	0.058	-							
	Age	0.267	-							
	T-stage	0.013	-							
	N-stage	0.027	0.012	3.227 (1.30-8.02)						
	HPV	0.079	-							
	Location PT	0.561	-							
	Tobacco usage	0.276	-							
DWI	ADC_GTV (cm^3)	0.046	-		0.262	-		0.071	-	
	ADC_median ($x10^3$ m^2/s)	0.038	-		0.049	-		0.769	-	
	ADC_Skewness	0.001	-		0.005	0.01	3.359 (1.18-9.54)	0.754	-	
	ADC_Kurtosis	<0.001	<0.001	3.957 (2.09-7.51)	0.029	-		0.065	-	
IVIM	D (xE3 mm^2/s)	0.019	0.019	0.015 (0.0004-0.498)	0.455	-		0.311	-	
	f (x10 mm^2/s)	0.955	-		0.199	-		0.447	-	
	D* (xE2 mm^2/s)	0.774	-		0.205	-		0.624	-	
DCE	DCE_{GTV} (cm^3)	0.065	-		0.138	-		0.364	-	
	K_{ep} (min^{-1})	0.815	-		0.777	-		0.598	-	
	K^{trans} (min^{-1})	0.430	-		0.927	-		0.015	0.015	2.617 (1.21-5.67)
	V_e	0.090	-		0.858	-		0.041	-	
^{18}F-FDG-PET	MATV (cm^3)	0.032	-		0.017	-		0.489	-	
	SUV_mean (Bq)	0.696	-		0.195	-		0.420	-	
	SUV_peak (Bq)	0.002	0.001	9.356 (2.51-34.9)	0.307	-		0.088	-	
	TLG (Bq x cm^3)	0.022	0.004	3.1E4 (25.5-3.8E6)	0.008	0.01	2.13 (1.21-3.74)	0.885	-	

* Univariable Cox-regression analysis ** Multivariable Cox-regression analysis

Multivariable analysis of functional imaging parameters per modality predicting distant metastasis. Significantly prognostic parameters are marked green.

cancers

MDPI

Table S6. Univariate and multivariate analysis predicting overall survival.

Overall survival

		PRETREATMENT			INTRATREATMENT			DELTA		
	n=57 patients	Univariate*	Multivariate**		Univariate*	Multivariate**		Univariate*	Multivariate**	
	Parameters	p-value*	p-value	HR (95%CI)	p-value*	p-value	HR (95%CI)	p-value*	p-value	HR (95%CI)
Clinical parameters	Gender	0.89	-							
	Age	0.92	-							
	T-stage	0.17	-							
	N-stage	0.17	0.03	3.373 (0.03-0.55)						
	HPV	0.02	0.01	0.123 (0.03-0.55)						
	Location PT	0.75	-							
	Tobacco usage	0.24	-							
DWI	ADC_GTV (cm^3)	0.02	0.02	1.059 (1.01-1.11)	0.01	0.001	1.074 (1.03-1.12)	0.74	-	
	ADC_median (xE^3 m^2/s)	0.65	-	-	0.69	-		0.99	-	
	ADC_Skewness	0.10	-	-	0.71	-		0.83	-	
	ADC_Kurtosis	0.98	-	-	0.33	-		0.86	-	
IVIM	Dt (xE3 mm^2/s)	0.61	-		0.23	-		0.10		
	F (xE^1 mm^2/s)	0.45	-		0.53	-		0.72		
	Dp (xE2 mm^2/s)	0.34	-		0.44	-		0.22		
DCE	DCE_{GTV} (cm^3)	0.02	0.02	1.054 (1.01-1.10)	0.02	0.02	1.057 (1.01-1.11)	0.44	-	
	K_{ep} (min^{-1})	0.85	-		0.25			0.11	0.001	0.00-0.56)
	K^{trans} (min^{-1})	0.74	-		0.39			0.55	-	
	V_e	0.46	-		0.94			0.54	-	
^{18}F-FDG-PET	MATV (cm^3)	0.00	0.001	1454.6 (11.54-1.8E5)	0.01	0.001	259.3 (5.76-1.17E4)	0.72	-	
	SUV_mean (Bq)	0.02	0.03	0.228 (0.06-0.89)	0.08	-		0.80	-	
	SUV_peak (Bq)	0.45	-		0.16	-		0.07	-	
	TLG (Bq x cm^3)	0.60	-		0.07	-		0.70	-	

* Univariable Cox-regression analysis ** Multivariable Cox-regression analysis

Multivariable analysis of functional imaging parameters per modality predicting adverse overall survival. Significantly prognostic parameters are marked green.

Table S7. Logistic-regression prognostic models of primary tumours

Logistic-regression prognostic models of primary tumours						
**Outcome**	**Model**	**Imaging time**	**Selected features**		**Selected Clinical features**	
Locoregional recurrence-free survival		Pretreatment	Intercept = -2.246	Slope	Intercept = -1.859	**Slope**
			ADC_kurtosis	0.211	AD_Ckurtosis	0.134
			SUV_peak	2.145	SUV_peak	1.945
		Intratreatment	Intercept = -		Intercept = -	
			-		-	
		Delta	Intercept = -0.471		Intercept = -0.244	
			ADCGTV	-0.558	Age	0.0008
			ADC_median	5.154	N-stage	0.709
			ADC_skewness	-0.041	ADC_skewness	-0.003
			D	0.030	f	-0.005
			f	-0.459	SUV_peak	-0.368
			DCE_GTV	0.222	TLG	-0.026
			Kep	-0.235		
			Ve	0.458		
			SUV_peak	-0.627		
			TLG	-0.075		
		Pre-/intratreatment/delta	Intercept = -2.173		Intercept = -1.804	
			PRE-ADC_kurtosis	0.317	PRE-ADC_kurtosis	0.235
			PRE-Kep	0.169	PRE-SUV_peak	1.444
			PRE-SUV_peak	1.470	Δ-ADC_skewness	-0.002
			Δ-ADC_skewness	0.011	Δ-SUV_peak	-0.15
			Δ-f	-0.036	Δ-TLG	-0.002
			Δ-SUV_peak	-0.191		
			Δ-TLG	-0.009		
Distant metastasis		Pretreatment	Intercept = -5.950		Intercept = -7.511	
			ADC_kurtosis	1.695	Gender	0.079
			D	0.971	Tobacco	-0.166
			SUV_peak	0.203	ADC_kurtosis	1.542
					PRE-D*	0.700
					PRE-SUV_peak	0.166
		Intratreatment	Intercept = -1.268		Intercept = -1.949	
			ADC_skewness	1.223	Gender	0.996
					HPV	0.149
					T-stage	-0.392
					ADC_skewness	1.362
		Delta	Intercept = -1.616		Intercept = -10.144	
			ADCGTV	-0.581	Gender	1.54
			ADC_kurtosis	-3.044	Age	0.104
			D	1.154	HPV	0.676
			Ve	0.648	Tobacco	-0.796
			SUV_mean	-0.114	N-stage	0.355
					ADC_GTV	-0.018
					ADC_skewness	0.0004
					ADC_Kurtosis	-4.846
					D	2.635
					Ve	1.001
					SUV_peak	-0.106
					SUV_mean	-0.151
			Intercept = -6.754		Intercept = - 5.950	

	Pre-/intratreatment/delta	PRE-ADC_kurtosis	1.392	PRE-ADC_kurtosis	1.277
		INRA-ADC_skewness	0.154	PRE-SUV_peak	0.017
		PRE-D*	0.333		
		PRE-SUVpeak	0.149		
Overall survival	Pretreatment	Intercept = -1.080		Intercept = -1.387	
		ADC_skewness	0.174	Gender	-0.685
		MATV	6.541	HPV	-3.196
		SUV_mean	-0.979	N-stage	0.908
				PRE-ADC_skewness	0.926
				PRE-D	-0.139
				PRE-f	-0.278
				PRE-MATV	8.64
				PRE-SUV_peak	-0.836
				PRE-SUV_mean	1.886
	Intratreatment	Intercept = -		Intercept = -1.178	
		-		HPV-status	-1.487
				N-stage	0.51
				INTRA-DCE_GTV	0.022
				INRA_MATV	2.243
	Delta	Intercept = -0.856		Intercept = -0.659	
		SUV_peak	-1.159	HPV-status	-1.444
				N-stage	0.532
				Δ-D	0.304
				Δ-SUV_peak	-0.099
	Pre-/intratreatment/delta	Intercept = -1.151		Intercept = -1.072	
		PRE-MATV	3.726	HPV-status	-1.369
		PRE-SUV_mean	-0.178	N-stage	0.158
				PRE-ADC_skewness	0.479
				PRE-MATV	5.163
				PRE-SUV_peak	-0.049
				PRE-SUV_mean	-0.312
				Δ-Kep	-0.027
				Δ-SUV_peak	-0.049

Table S7. Lasso logistic-regression in pretreatment, intratreatment and delta-imaging features and combined for predicting LRFFS, DMFS and OS. Lambda = median cross-validated lambda with 10 folds and 500 repeats. Intercept and slope were presented for each model.

Table S8. Cox-regression prognostic models of primary tumours

Cox-regression prognostic models of primary tumours					
**Outcome**	**Model Imaging time**	**Selected features**	**Hazard ratio**	**Selected Clinical + imaging features**	**Hazard ratio**
**Locoregional recurrence-free survival**	**Pretreatment**	PRE-ADC_kurtosis	1.101	PRE-ADC_kurtosis	1.063
		PRE-Kep	1.813	PRE-Kep	1.504
		PRE-SUV_peak	3.858	PRE-SUV_peak	3.532
	**Intratreatment**	-		-	
		-		-	
		-		-	
		-		-	
	**Delta**	Δ-ADC_GTV	0.682	Age	1.004
		Δ-ADC_median	12.472	N-stage	1.39
		Δ-ADC_skewness	0.982	ADC_skewness	0.985
		Δ-D	0.887	f	0.967
		Δ-f	1.298	SUV_mean	0.894
		Δ-Kep	0.735	SUV_peak	0.783
		Δ-SUV_mean	0.770		
		Δ-SUV_peak	0.684		
	**Pre-/intratreatment/delta**	PRE-ADC_kurtosis	1.235	PRE-ADC_kurtosis	1.215
		PRE-D	0.753	PRE-D	0.821
		PRE-Kep	2.321	PRE-Kep	2.159
		PRE-SUV_peak	2.004	PRE-SUV_peak	2.039
		Δ-ADC_skewness	0.971	Δ-ADC_skewness	0.974
		Δ-f	0.966	Δ-f	0.978
		Δ-SUV_mean	0.903	Δ-SUV_mean	0.916
		Δ-SUV_peak	0.818	Δ-SUV_peak	0.834
**Distant metastasis**	**Pretreatment**	PRE-ADC_skewness	1.568	PRE-ADC_skewness	1.151
		PRE-ADC_kurtosis	1.952	PRE-ADC_kurtosis	1.901
		PRE-SUV_peak	1.646	PRE-SUV_peak	1.241
		PRE-TLG	3.923		
	**Intratreatment**	INTRA-ADC_skewness	1.640	Gender	1.444
		INTRA-TLG	1.006	Tobacco use	0.650
				T-stage	0.565
				INTRA-ADC_skewness	1.640
				INTRA-MATV	28.602
				INTRA-TLG	1.006
	**Delta**	Δ-ADCGTV	0.842	Gender	1.383
		Δ-ADC_kurtosis	0.609	Tobacco use	0.577
		Δ-Ktrans	1.404	T-stage	0.882
		Δ-Ve	1.164	N-stage	1.600
		Δ-SUV_peak	0.965	Δ-ADC_kurtosis	0.820
				Δ-Ktrans	1.338
				Δ-Ve	1.077
				Δ-SUV_peak	0.947
		Δ-ADC_kurtosis	1.822	PRE-ADC_skewness	1.063

cancers

MDPI

	Pre-/intratreatment/delta	PRE-SUV_peak	1.063	PRE-ADC_kurtosis	1.869
				PRE-SUV_peak	1.151
Overall survival	Pretreatment	PRE-ADC_skewness	1.025	HPV	0.237
		PRE-MATV	164.50	N-stage	1.153
		PRE-SUV_mean	0.419	PRE-ADC_skewness	1.823
				PRE-MATV	125.25
				PRE-SUV_mean	0.439
				PRE-SUV_peak	1.810
	Intratreatment	INTRA-ADCGTV	1.014	HPV	0.604
		INTRA-MATV	1.802	ADC_GTV	1.014
				MATV	1.802
	Delta	Δ-D	1.692	HPV	0.37
		Δ-D*	0.939	N-stage	1.576
		Δ-Kep	0.842	D	1.054
		Δ-SUV_peak	0.914	Kep	0.842
				SUV_peak	0.915
	Pre-/intratreatment/delta	PRE-MATV	35.492	HPV-status	0.485
		PRE-SUV_mean	0.614	PRE-ADC_skewness	1.250
				PRE-MATV	58.848
				PRE-SUV_mean	0.593
				Δ-Kep	0.9799
				Δ-SUV_peak	0.977

Table S9. Lasso cox-regression in pretreatment, intratreatment and delta-imaging features and combined for predicting LRFFS, DMFS and OS. The selected features are presented with the hazard ratios. Lambda = median cross-validated lambda with 10 folds and 500 repeats.

cancers MDPI

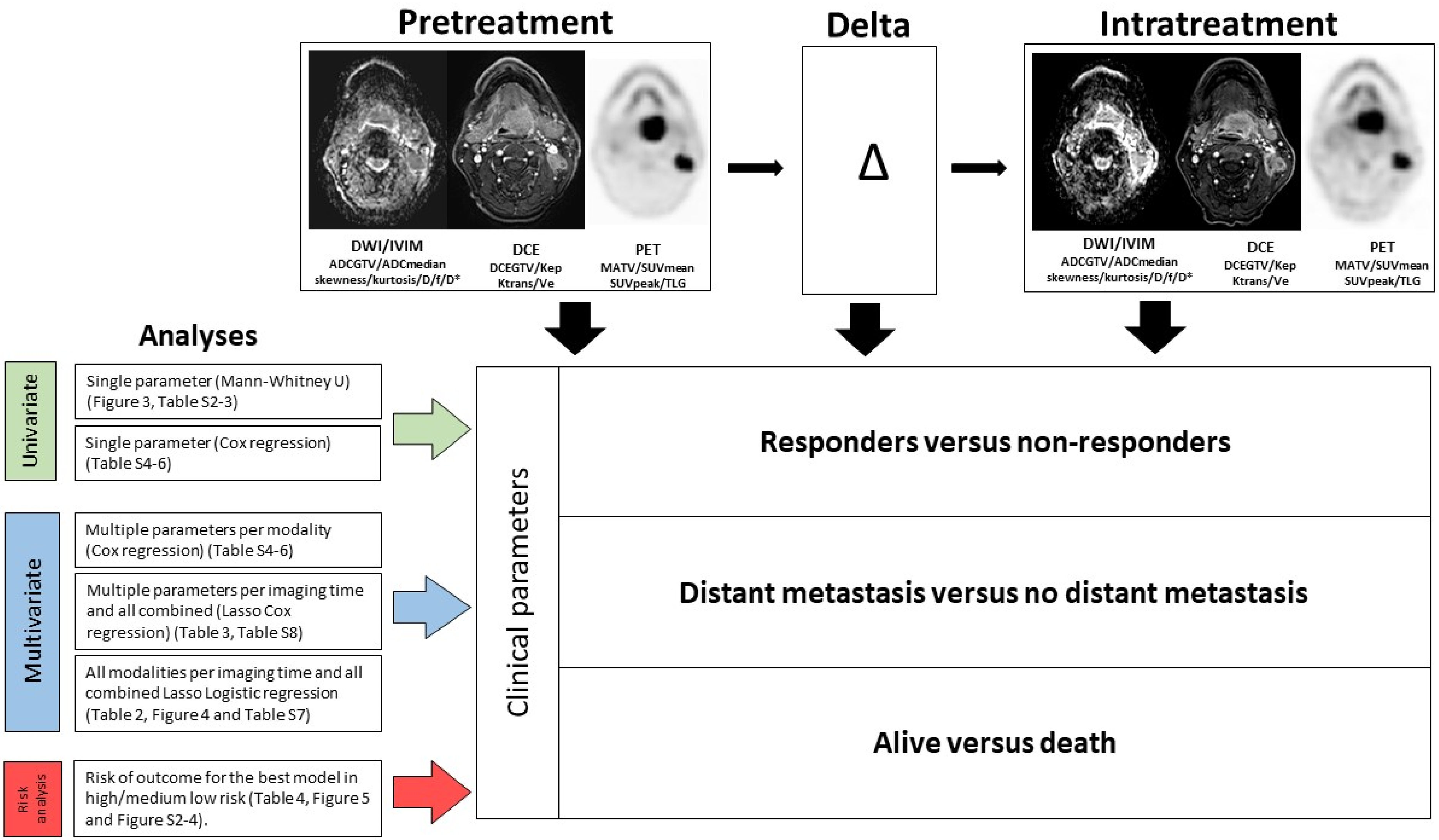

Figure S1. Overview of performed analysis in current study with the univariate/multivariate and risk analysis for primary tumors in pretreatment, delta and intratreatment imaging times and all three combined.

cancers

MDPI

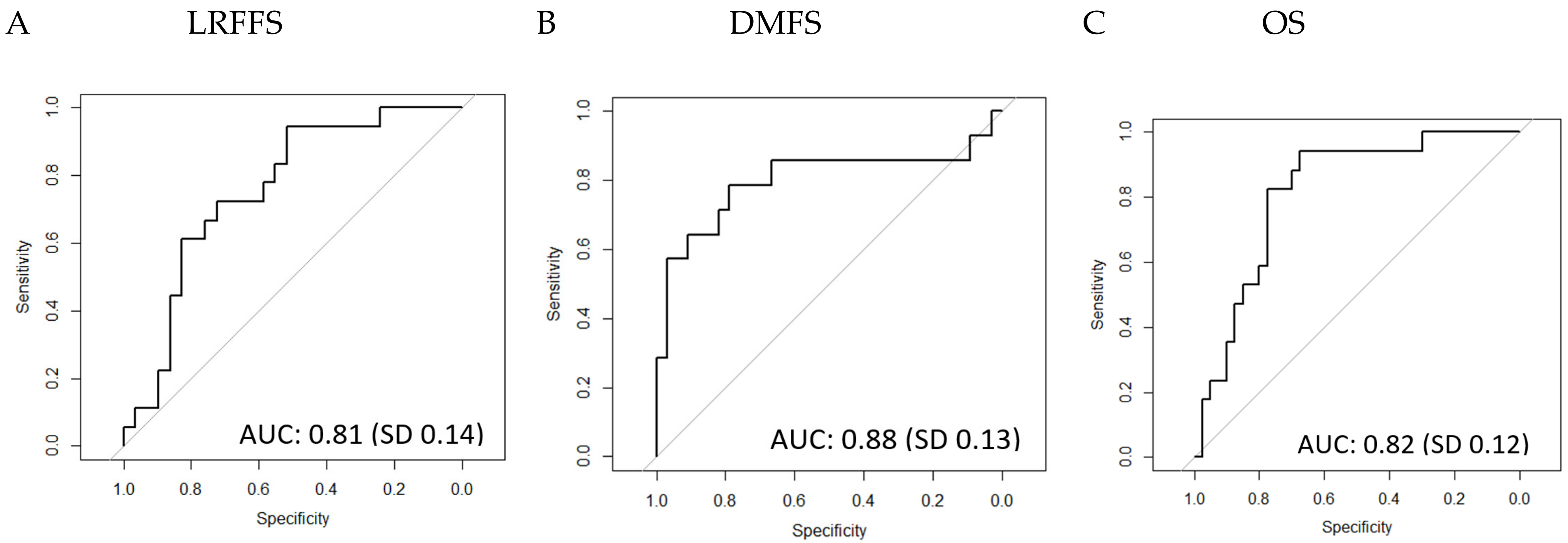

Figure S2. The area under the curves for the high risk group of patients, using the best (A) locoregional recurrence-free survival (LRFFS), (B) distant-metastasis-free survival (DMFS) and (C) overall survival (OS)

cancers

MDPI

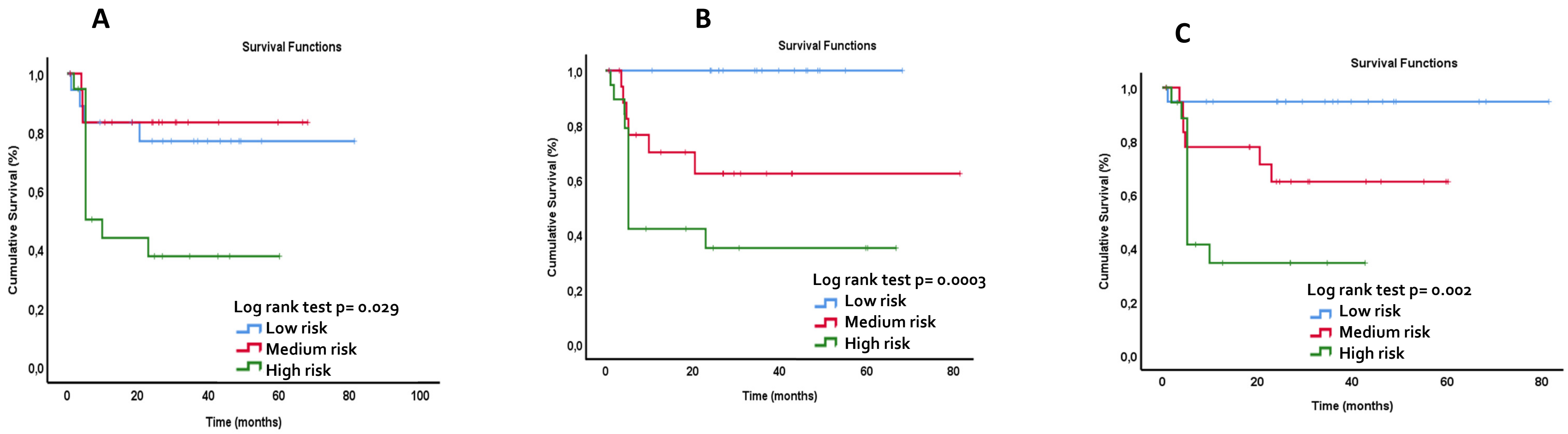

Figure S3. Kaplan meier curves with logrank test for locoregional recurrence-free survival divided in high/medium/low risk patient groups for pretreatment (A), intratreatment (B) and delta parameters (C).

cancers MDPI

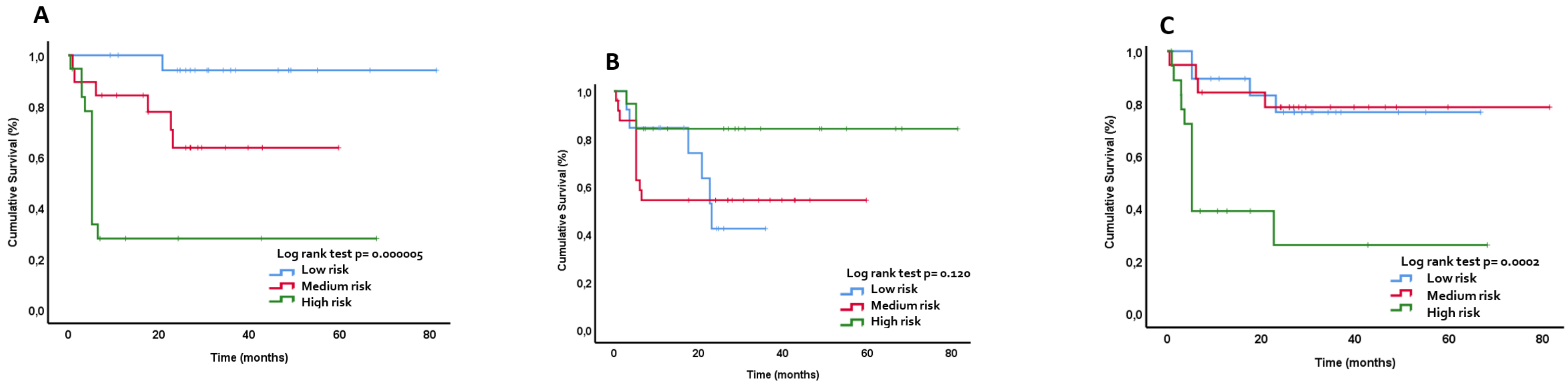

Figure S4. Kaplan Meier curves with logrank test for distant metastasis-free survival divided in high/medium/low risk patient groups for pretreatment (A), intratreatment (B) and delta parameters (C).

cancers

MDPI

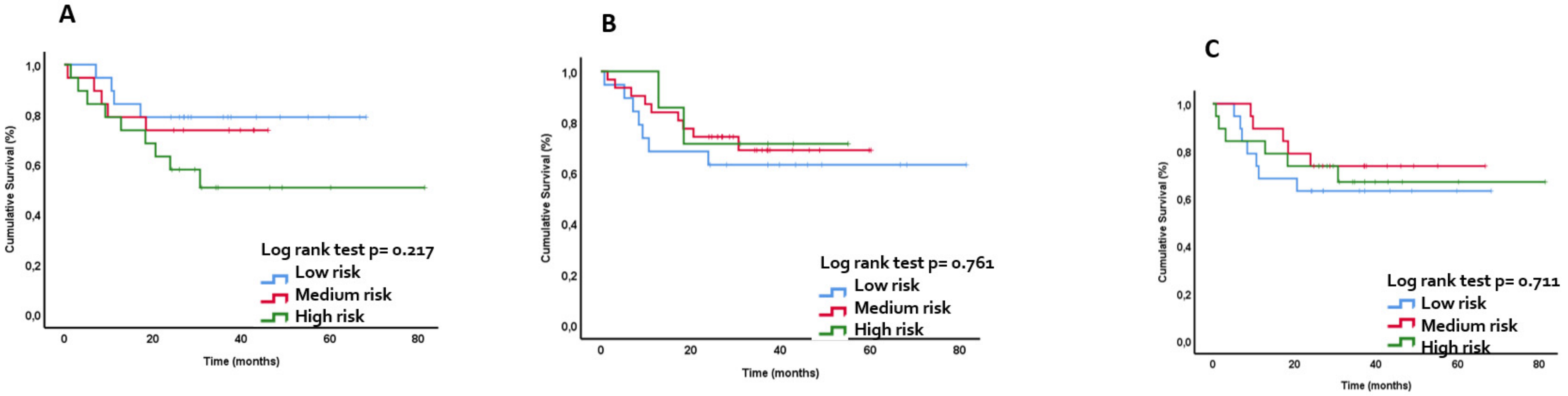

Figure S5. Kaplan meier curves with logrank test for overall survival divided in high/medium/low risk patient groups for pretreatment (A), intratreatment (B) and delta parameters (C).
